# Exploring genetic diversity in genome-wide association studies and polygenic risk scores in the admixed Brazilian population

**DOI:** 10.1590/1678-4685-GMB-2025-0235

**Published:** 2026-08-24

**Authors:** Miriam Coelho Molck, Luciano Henrique Braz dos Santos, Thais C. de Oliveira, Iscia Lopes-Cendes

**Affiliations:** 1 Universidade Estadual de Campinas, Faculdade de Ciências Médicas, Departamento de Genética Médica e Medicina Genômica, Campinas, SP, Brazil.; 2 Brazilian Institute of Neuroscience and Neurotechnology (BRAINN), Campinas, SP, Brazil.

**Keywords:** Brazil, population admixture, precision medicine, GWAS, PRS

## Abstract

The Brazilian population has a complex genetic background resulting from admixture of Native American, African, and European ancestries. This review examines how such diversity influences the findings of genome-wide association studies and the performance of polygenic risk score. It also highlights the role of admixture in elucidating the genetic basis of complex diseases, and addresses key methodological challenges, including population stratification and limited representation in genomic databases. Furthermore, it evaluates the transferability of risk models developed in other populations. The review underscores the need for inclusive research strategies to improve the accuracy and relevance of genomic predictions for precision medicine worldwide.

## Introduction

Brazil’s genetic landscape has resulted from 500 years of admixture among Native American (NAM), African (AFR), and European (EUR) populations. However, the proportion of these ancestries varies significantly across different regions. This reflects distinct historical migration patterns (Kehdy et al., 2015). The Southern region predominantly exhibits EUR ancestry (~77%). The Northeast shows higher AFR ancestry (~27%). The North has the greatest NAM contribution (~32%) (Rodrigues de Moura et al., 2015). Compared with other Latin American and Caribbean (LAC) populations, Brazilians generally present lower NAM ancestry (~12%), higher AFR background (~20-27%), and greater regional heterogeneity (Ruiz-Linarés, 2015). Despite recent developments, the number of local genomic studies remains limited. This limitation, together with Brazil’s extensive ancestral variability, poses a significant challenge for research and genomics applications in medicine. As a result, many population-specific variants found in the Brazilian population are absent from global databases, which may hinder accurate clinical interpretation of genetic findings (Mostafavi et al., 2020).

**Figure 1 -:**
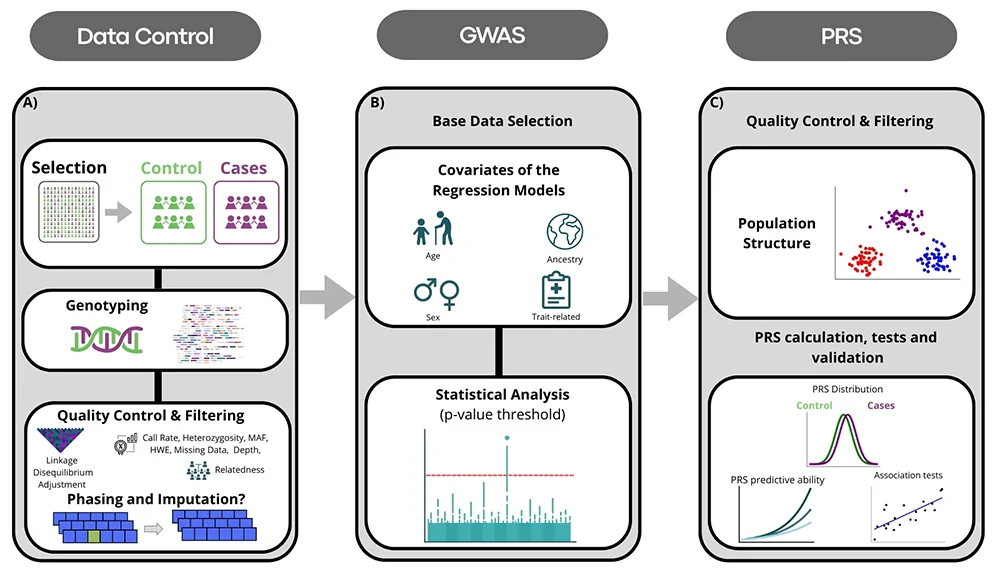
Schematic representation of the workflow for conducting genome-wide association studies (GWAS) and polygenic risk score (PRS) analyses. (A) An example of data control is selecting samples stratified into case and control groups. Quality control steps were applied to remove errors and biases, including checks for call rate, heterozygosity, minor allele frequency (MAF), Hardy-Weinberg equilibrium (HWE), missing data, sequencing depth, linkage disequilibrium (LD) adjustment, and relatedness. When required, phasing and imputation are performed to infer the untyped variants. (B) GWAS begins by integrating covariates of the regression models, such as age, sex, ancestry, and trait-related information, followed by statistical testing of genetic variants across the genome. Associations are evaluated using predefined significance thresholds. (C) PRS analyses are conducted using GWAS summary statistics as base data and independent individual-level genotypic and phenotypic information as target data. Quality control (QC) ensures adequate variant overlap across datasets and corrects for population structure. PRS calculation includes distribution analyses between cases and controls, evaluation of predictive ability, and validation through association tests and cross-validation in independent cohorts.

Genomic studies in LAC populations have revealed complex ancestry substructures and marked regional heterogeneity, highlighting the value of incorporating local ancestry data to refine the identification of genetic variants associated with specific phenotypes in admixed populations, where linkage disequilibrium (LD) patterns may differ across ancestral backgrounds (Martin et al., 2017; Secolin et al., 2019). This genetic architecture manifests as unique haplotype structures and ancestry-specific LD patterns shaped by distinct admixture processes and LD decay rates (Zhou et al., 2017). Therefore, genetic associations, including single-nucleotide polymorphisms (SNPs) and structural variants identified in non-admixed populations, require rigorous validation within the Brazilian genetic context to reduce spurious associations and cross-ethnic false positives and to ensure the clinical validity of these findings (Kaibara et al., 2021). Additionally, allelic association studies, including genome-wide association studies (GWAS) and polygenic risk scores (PRS) (Figure 1), depend on haplotype structures because flanking alleles mark core causal variants (da Cruz et al., 2022). However, local ancestry inference may result in inaccuracies in predicting regional ancestry proportions due to genetic drift, natural selection, and methodological limitations (Secolin *et al.*, 2019).

These methodological challenges have been central to the design of major genomic studies in Brazil. Key initiatives include the Brazilian Initiative on Precision Medicine (BIPMed) (Rocha et al., 2020), Health, Welfare, and Aging (SABE) (Lebrão et al., 2019), Brazilian Online Archive of Mutations (ABraOM) (Naslavsky et al., 2022), South American Epidemiology and Genetics Cohort (EPIGEN-Brasil) (Lima-Costa et al., 2015), and Single-Institution Genomic Database of the Southeastern Brazilian Population (SELAdb) (Lerario et al., 2020). Over the past decade, these efforts culminated in the development of the Genomas Brasil program. This program aims to map the country’s genomic diversity across several domains. These include population genomics (DNA of Brazil (DNABr) project and Brazil Genomics Map), rare diseases (Brazilian Rare Genomics Project (BRGP)), oncology (Onco-Genomas Brasil), and cardiovascular disease (Brazilian National Network of Cardiovascular Genomics (RENOMICA)) (Brasil, 2020a). These national initiatives and the growing interest in genomic medicine have facilitated the development of analytical frameworks tailored to the Brazilian population, including the establishment of genomic databases and the generation of datasets to support GWAS and PRS analyses (Novembre et al., 2022).

Genome-wide association studies (GWAS) identify genetic variants linked to complex traits by scanning the genome, exome, or SNP arrays (Visscher et al., 2017). Polygenic risk scores (PRS) represent the cumulative effects of many genetic variants, each contributing a small fraction to the overall risk of a trait or disease. PRS use GWAS findings by weighting variants according to their estimated effect sizes and summing these contributions. They can be applied alone or integrated with non-genetic variables such as age, sex, environmental exposures, ancestry, or clinical parameters (Torkamani et al., 2018). The clinical utility of PRS is primarily constrained by the sample size and ancestral diversity of GWAS. Major limitations include: (a) insufficient sample sizes, which reduce statistical power to detect variants with small effects; (b) imprecise estimation of SNP effect sizes, leading to inaccurate weighting and reduced predictive performance; (c) ancestry bias, driven by the overrepresentation of EUR populations in GWAS, which limits transferability to admixed populations; and (d) methodological constraints related to trait architecture, such as low SNP-based heritability, which can affect the reliability of genetic predictions (Choi et al., 2020).

PRS predictive performance declines significantly across ancestries. In admixed populations such as those in Brazil, this reduction reflects differences in linkage disequilibrium patterns, allele frequencies, and population-specific interactions, including gene-environment and epistatic effects (Martin et al., 2019). Understanding this population-specific genetic architecture is essential to improve clinical accuracy and support equitable health outcomes (Barreiro et al., 2024). PRS derived from GWAS conducted in European (EUR) populations may lack key information for admixed individuals of LAC ancestry, even when a substantial EUR component is present (Martin *et al.*, 2019). This limited transferability reduces predictive accuracy in admixed populations and contributes to disparities in genomic medicine. These gaps widen the precision medicine divide and hinder progress toward equitable health outcomes (Popejoy and Fullerton, 2016).

Building on these challenges, the American Society of Human Genetics (ASHG) has emphasized the inclusion of underrepresented populations in genomic research to improve the accuracy of PRS across ancestries (Sirugo et al., 2019). For LAC populations, the development of self-referential genomic datasets is therefore a critical step toward overcoming existing biases, enabling more accurate risk estimation, and advancing personalized medicine (de Oliveira et al., 2023; de Oliveira and Lopes-Cendes, 2025).

This systematic review aims to: (i) summarize GWAS and PRS studies in Brazilian individuals and multiethnic cohorts including Brazilian participants; (ii) examine how genetic diversity and admixture influence disease susceptibility and risk; (iii) evaluate methodological limitations, including population stratification; and (iv) assess opportunities and barriers for the implementation of genomic medicine in Brazil.

## Material and Methods

A comprehensive literature search was conducted by two independent reviewers in PubMed to identify publications on GWAS and PRS in Brazilian cohorts. The search strategy combined key terms including “Brazil,” “Brazilian,” “Latin America,” and “Admixture” with “GWAS,” “Genome-Wide Association Study,” “PRS,” and “Polygenic Risk Score.” Additionally, disease-specific terms were incorporated, such as “Cardiovascular Diseases*,” “Atrial Fibrillation,” “Stroke,” “Sickle Cell Disease,” “Cancer*,” “Osteosarcoma,” “Oral Cavity Cancer,” “Oropharyngeal Cancers,” “Breast Cancer,” “Pancreatic Cancer,” “Kidney Cancer,” “Metabolic Diseases*,” “Overweight,” “Obesity,” “Body Mass Index,” “Type 2 Diabetes,” “Neurological and Psychiatric Disorders*,” “Age-related cognitive decline,” “Brain Morphometry,” “Epilepsy,” “Genetic Generalized Epilepsies,” “Machado Joseph Disease,” “Parkinson’s Disease,” “Attention-Deficit/Hyperactivity Disorder,” “Schizophrenia,” “Glaucoma,” “Primary Angle-Closure Glaucoma,” and “Coronavirus disease 2019*” or “COVID-19.”

To ensure a comprehensive search, we queried the GWAS Catalog (GWAS Catalog, 2025), a curated repository of published GWAS and their association results, and the Polygenic Score (PGS) Catalog (PGS Catalog, 2025), an open database that provides polygenic scores and associated metadata for accurate application and evaluation.

**Figure 2 -:**
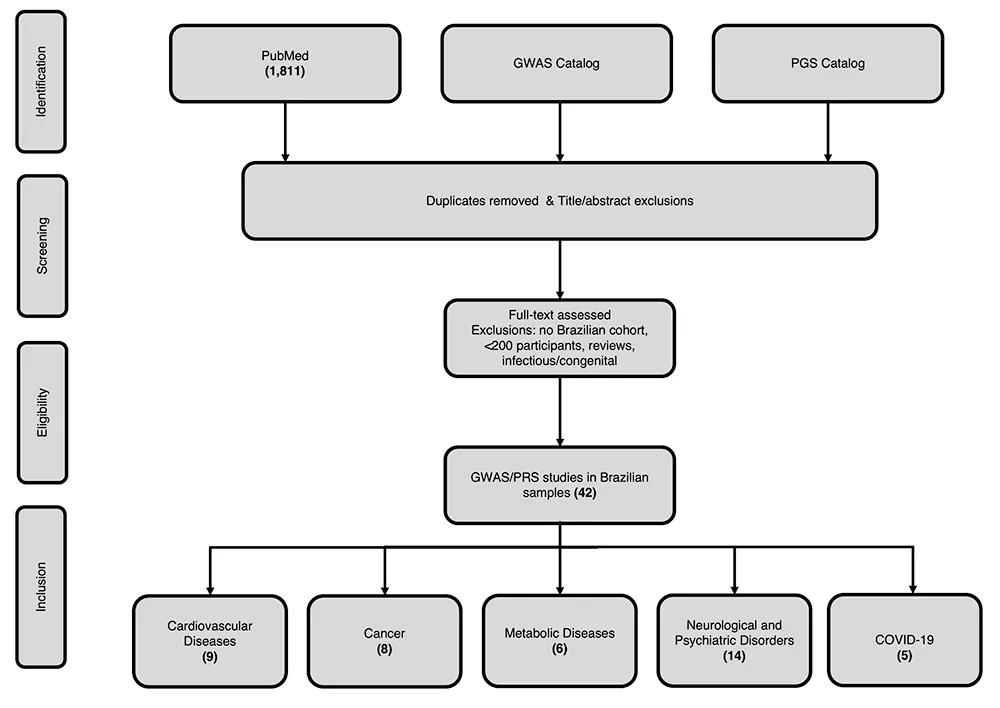
Flow diagram illustrating the steps of the systematic review process that led to the selection of 42 genome-wide association studies and polygenic risk score studies including Brazilian cohorts.

Studies were included if they met the following criteria: (i) published between 2010 and 2025, (ii) written in English, (iii) had full-text available, and (iv) included Brazilian participants or populations of Brazilian ancestry. The exclusion criteria were as follows: (i) absence of Brazilian participants or genomic data from Brazilian populations; (ii) fewer than 200 participants in the GWAS or PRS analyses; (iii) review articles, editorials, or conference abstracts; and (iv) exclusive focus on infectious diseases other than COVID-19 and on or congenital syndromes. Two independent reviewers screened the titles and abstracts for eligibility, and disagreements were resolved through discussion or consultation with a third reviewer. Full-text articles that met the inclusion criteria were retrieved and evaluated for final inclusion (Figure 2).

The data extracted from the 42 eligible studies included the study design, population characteristics, ancestry composition, number of Brazilian individuals, geographic region or state of origin, significant SNPs identified, and key findings related to GWAS and PRS analyses (Table S1).

## Results and Discussion

**Figure 3 -:**
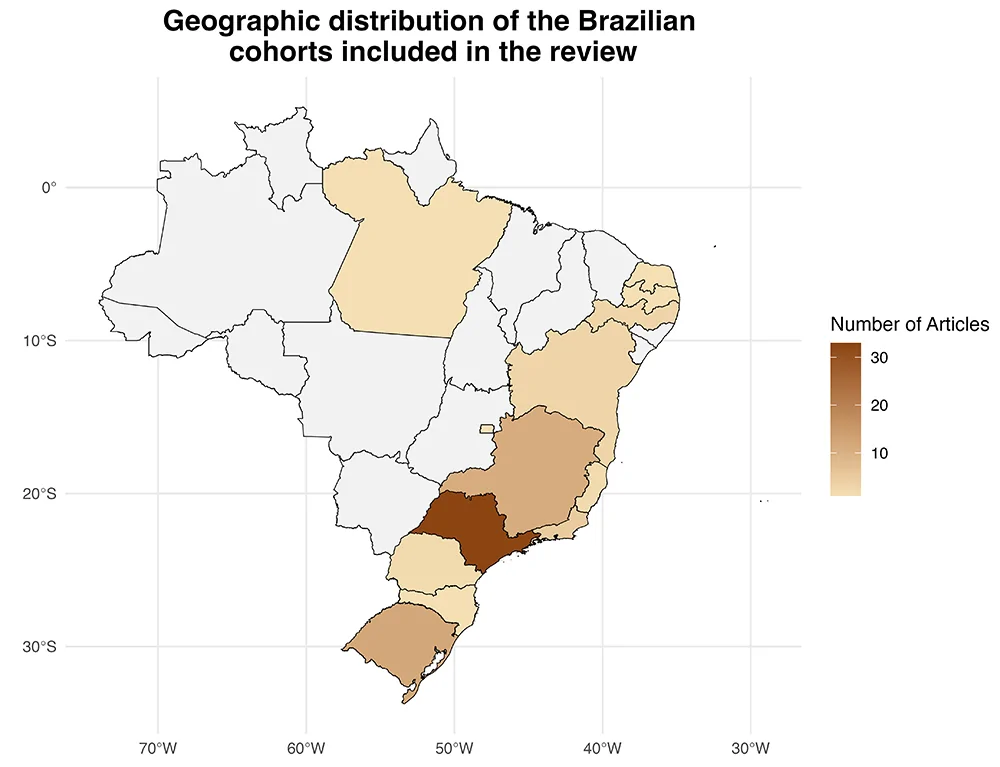
Geographic distribution of the Brazilian cohorts included in the 42 studies covered in this review. A color code was used to depict the proportion of studies in each Brazilian state. There was marked geographic inequality in the distribution of these studies, with most cohorts originating from São Paulo (30 studies, 71.4%).

Using the methodology described, 42 studies were identified and are listed chronologically within specific categories in Table S1. The state of São Paulo contributed 30 studies (71.4%), illustrating the unequal geographic distribution of genomic research within Brazil (Figure 3). São Paulo accounts for approximately 21.8% of the Brazilian population and, as the most populous and economically developed state, attracts individuals from diverse regions (IBGE, 2022). However, this concentration raises important concerns, as São Paulo harbors a relatively higher proportion of Asian (ASI) ancestry compared with other regions of Brazil, which may introduce regional biases in genetic representation (Kehdy et al., 2015). Consequently, reliance on São Paulo-centered research may overlook the diverse genomic profiles of populations in the North and Northeast, undermining the transferability of clinical findings and limiting our understanding of Brazil’s broader genomic composition (Nunes et al., 2025).

The application of GWAS and PRS methodologies in Brazilian populations comprises a wide range of health conditions, reflecting the nation’s unique epidemiological profile and the potential of genomic approaches to address population-specific disease burden. This review organizes genomic findings by major disease categories* to assess the influence of genetic diversity and admixture on disease susceptibility and risk. These categories include cardiovascular diseases*, the leading cause of morbidity and mortality in Brazil; cancer*, comprising various malignancies with distinct genetic architectures; metabolic diseases*, such as overweight, obesity, and type 2 diabetes; neurological and psychiatric disorders*, characterized by complex genetic phenotypes influenced by population-specific variants; and coronavirus disease 2019* (COVID-19*), a global pandemic with population-specific genetic determinants affecting disease severity and susceptibility.

### Cardiovascular diseases

Cardiovascular diseases are the leading cause of morbidity and mortality in Brazil, accounting for approximately 30% of all annual deaths (Oliveira et al., 2022). The complex genetic architecture underlying these conditions, combined with environmental and lifestyle factors, makes them particularly suitable for genomic investigations in admixed populations (Kehdy et al., 2015). 

*Atrial Fibrillation* (AF) is the most common cardiac arrhythmia, affecting more than 33 million individuals worldwide, and has a complex heritable component (Chugh et al., 2014). During the last decade, GWAS have identified 13 genetic loci associated with AF in EUR and one ASI-specific AF locus, of which a region near the *PITX*2 gene showed the strongest association (Sinner et al., 2014). Christophersen et al. (2017) identified 12 additional AF susceptibility loci in a multi-ancestry GWAS meta-analysis within the AFGen Consortium, including 33 studies with 18,398 individuals with AF and 91,536 controls, one of which was a Brazilian cohort (187 cases and 550 controls). The newly identified loci implicated genes involved in cardiac electrical and structural remodeling, including *PITX2*, *ZFHX3*, *PRRX1*, *SOX5*, and *TBX5*, thereby doubling the number of known AF loci and providing new insights into the molecular basis of AF. A significant association between AF and variants in *PITX2* was found in individuals of AFR-NAM (rs6843082), in individuals of ASI ancestry (rs2723334) and in individuals of AFR-NAM and EUR ancestry (rs2129977). Roselli et al. (2018) extended these findings with a large, multi-ancestry GWAS including 65,446 individuals with AF from over 50 studies, including a Brazilian cohort (568 cases and 1,096 controls). In total, 97 loci were significantly associated with AF (67 novel in the combined ancestry analysis and three in a EUR-specific analysis). Transcriptome-wide analyses detected 57 AF-associated genes, 42 of which overlapped with GWAS loci and were enriched in cardiac pathways. Overall, this study increased the number of known AF risk loci by more than threefold. 

Beyond AF case-control GWAS, several studies have investigated electrocardiographic traits related to atrial conduction and arrhythmia susceptibility. Ntalla et al. (2020) performed a multi-ancestry GWAS meta-analysis of the PR interval, an electrocardiographic marker representing atrial and atrioventricular conduction, in 293,051 individuals. The analysis included 40 studies from the CHARGE consortium, containing a Brazilian cohort (n = 485). A total of 202 loci were identified (141 novel loci, including 12 candidate genes with functional roles in cytoskeletal assembly). PRS analyses indicated that common variants in Mendelian cardiovascular disease genes contribute to population-level variation in the PR interval. These data collectively explained ~62% of the trait-related heritability and suggested that genetic determinants of the PR interval may identify distinct pathophysiological mechanisms underlying AF. Thériault et al. (2022) conducted a GWAS meta-analysis of premature atrial contraction (PAC) frequency using ambulatory cardiac monitoring data from 4,831 individuals across five population-based cohorts, including 405 cases from the Baependi Heart Study (BHS) from Brazil. Because of the limited sample sizes for other ancestries, only individuals of EUR origin were included, an important limitation because it excludes genetic variants specific to AFR or NAM backgrounds prevalent in the Brazilian population. Six novel loci were identified, including genes associated with inherited arrhythmic syndromes and cardiomyopathies (e.g., *SCN5A*). In contrast, several established AF risk loci, including *PITX2*, were not associated with PAC, suggesting that PAC frequency and AF share some, but not all, genetic determinants. Young et al. (2023) performed multi-ancestry GWAS meta-analyses across 14 studies (32 ancestry-specific sub-studies) and 23 studies (40 ancestry-specific sub-studies), also from the CHARGE consortium, including 518 cases from the Bambuí Brazil Cohort Study of Aging (BAMBUI). The study evaluated the QRS-T angle (QRS-Ta), an electrocardiographic marker that measures the deviation between the QRS complex (ventricular depolarization) and the T wave (ventricular repolarization). Sixty-one loci (58 novel) associated with spatial QRS-Ta and 11 loci associated with frontal QRS-Ta were identified, supporting spatial QRS-Ta as a genetically informed marker of arrhythmogenesis and highlighting its potential value for future PRS development. 

*Stroke* is the second leading cause of death worldwide and a leading cause of long-term disability (WHO, 2008). *Ischemic stroke* (IS), the predominant subtype, is caused by arterial occlusion in the brain and is a major comorbidity of *Sickle Cell Disease* (SCD), which affects more than 13 million individuals globally (Piel et al., 2017). Carneiro-Proietti et al. (2018) performed GWAS genotyping using a customized Transfusion Medicine Array in a large multicenter Brazilian SCD cohort. A total of 2,795 participants were enrolled at six sites between 2013 and 2015. Hemoglobin (Hb) SS was the most common SCD genotype (70.7%), and the SNP data revealed substantial genetic admixture within the cohort. Ibanez et al. (2022) investigated the genetic architecture of early neurological instability, defined as the change in the National Institutes of Health Stroke Scale (NIHSS) score within 6 hours and at 24 hours after stroke onset. The multi-ancestry GWAS meta-analysis included 5,876 individuals from seven countries (Spain, Finland, Poland, the United States, Costa Rica, Mexico, and Korea), together with a Brazilian cohort from the Brazilian Institute of Neuroscience and Neurotechnology-University of Campinas (BRAINN-UNICAMP) participating in MEGASTROKE (Malik et al., 2018). Common genetic variants explained 8.7% of the phenotypic variance in the NIHSS score at 24 h. Expression quantitative trait loci (eQTL) mapping and summary data-based Mendelian randomization (SMR) indicated that *ADAM23*, *GRIA1*, *GNPAT*, and *ABCB5* were associated with acute IS. Mishra et al. (2022) performed a cross-ancestry GWAS meta-analysis including 110,182 individuals with IS (five ancestries, 33% non-EUR) and 1,503,898 controls, including a Brazilian cohort (Joinville Biobank). Associations were detected at 89 independent loci (61 novel loci). Cross-ancestry fine-mapping, in silico mutagenesis analysis, transcriptome-wide and proteome-wide association analyses revealed two putative causal genes (*SH3PXD2A* and *FURIN*) and variants located in two additional genes (*GRK5* and *NOS3*). Using a three-pronged approach, genetic evidence for putative drug effects was identified in six genes (*F11*, *KLKB1*, *PROC*, *GP1BA*, *LAMC2*, and *VCAM1*). PRS derived from cross-ancestry and ancestry-specific stroke GWAS were evaluated in an independent cohort of 52,600 clinical trial participants of EUR, East Asian (EAS), and AFR ancestry with cardiometabolic disease. These PRS strongly predicted IS independently of clinical risk factors. Earley et al. (2023) conducted the largest GWAS of IS to date, including 1,333 individuals with SCD from Brazil (178 cases and 1,155 controls). Fourteen significant loci were identified, including *ADAMTS2* and *CDK18*, which have been associated with IS in individuals aged < 65 years without SCD. A meta-analysis of independent SCD cohorts identified additional significant variants, such as rs1209987 (*TBC1D32*), rs188599171 (*CUX1*), rs77900855 (*BTG1*), and rs141674494 (*VPS13C*), supporting a multivariate model of early IS risk and suggesting a shared genetic architecture between individuals with and without SCD.

### Cancer

Cancer is the second leading cause of death in Brazil, with an estimated 704,000 new cases diagnosed annually (Santos et al., 2023). In admixed populations, genetic heterogeneity arising from diverse ancestral contributions may influence cancer predisposition, tumor biology, and treatment response, potentially limiting the transferability of genomic findings derived predominantly from EUR populations (Thomas and Peters, 2025). 

*Osteosarcoma* is the most common primary malignant bone tumor in children and young adults (Mirabello et al., 2011). Savage et al. (2013) conducted an international, multi-institutional, multi-stage GWAS including 941 osteosarcoma cases and 3,291 cancer-free adult controls of EUR ancestry, with Brazilian individuals included in the replication stage (247 cases). A fixed-effect meta-analysis identified three significant loci: rs1906953, located in the *GRM4* gene, and rs7591996 and rs10208273, both located in a gene desert on chromosome 2p25.2. Mirabello *et al.* (2015) analyzed clinical outcome data from 935 osteosarcoma cases from the previous GWAS (Savage *et al.* 2013) and four independent replication cohorts, including Brazilian participants (107 cases). An SNP (rs7034162) in the *NFIB* gene was significantly associated with metastasis in patients of EUR, AFR, and Brazilian ancestry. A transposon mouse model identified *NFIB* as a novel metastasis-susceptibility gene in osteosarcoma, in which its inactivation or reduced expression drives metastatic progression.

*Oral cavity* (OC) and *oropharyngeal cancers* (OPC) are primarily linked to tobacco and alcohol use, although infection with human papillomavirus (HPV) also plays an important role. The proportion of HPV-related OPC varies widely, approximately 60% in the United States, 30% in Europe, and lower rates in South America (~13-18%) (López et al., 2014). Genetic factors have been associated with susceptibility to OC and OPC (McKay et al., 2011). Lesseur et al. (2016) genotyped 13,107 individuals from 12 epidemiological studies across EUR and the Americas (including 986 cases and 1,135 controls from Brazil). Eight loci (seven novel) were identified, including a strong signal within the HLA region, which was narrowed to a class II haplotype. Combined OC and OPC were associated with loci at *HLA-DQB1*, *LHPP*, and *OR52N2/TRIM5*; OC alone was associated with two new loci (*GPN1* and *LAMC3*), as well as with known cancer loci (*CDKN2B-AS1* and *CLPTM1L*). Supervised ancestry analysis showed that more than 90% of the participants were predominantly of EUR ancestry, with moderate admixture observed in the LAC cohorts.

*Breast cancer* (BC) is the most common malignancy among women worldwide and is highly heterogeneous (Sung et al., 2021). Single-nucleotide polymorphisms (SNPs) located in long noncoding RNA (lncRNA) loci play an important role in BC susceptibility; however, little is known about their role in Brazilian cohorts. Mathias et al. (2023) were the first to demonstrate the association of four lncRNA-SNPs (rs4415084, rs7716600, and the rs3803662/rs4784227 haplotype) with increased BC risk in Brazilian samples (291 cases and 370 controls) by integrating genome-wide association study (GWAS) findings with The Cancer Genome Atlas (TCGA) expression data, emphasizing the need to expand research in this area. PRS analyses for BC have clinical relevance in risk prediction. Based on this, Barreiro et al. (2024) evaluated the transferability of a BC PRS composed of 313 risk variants (313-PRS) in two whole-genome-sequenced Brazilian cohorts (1,606 cases in total, SABE, n = 753; BRGP, n = 853) of tri-hybrid admixed ancestry (EUR, AFR, and NAM) and compared them with the UK Biobank (UKBB, n = 264,307) reference. Although the Brazilian cohorts had a substantial EUR component, with allele frequencies and LD patterns broadly similar to those of EUR populations, the 313-PRS distribution was inflated relative to the UKBB. This suggests a potential overestimation of risk if calibrated solely on EUR data and assuming that these cohorts have PRS distributions similar to those of the UKBB. Nevertheless, case-control analyses demonstrated predictive power comparable to that of UKBB-EUR samples, highlighting the cohort dependence of performance metrics despite similar ancestries and the need for more diverse populations for PRS calibration.

*Pancreatic and kidney cancers* are among the most lethal malignancies worldwide, particularly in Western populations (Bray et al., 2018). Genetic factors substantially contribute to their etiology, and successive GWAS have investigated the role of common low-penetrance variants in disease heritability (Gentiluomo et al., 2022). Lu et al. (2021) analyzed one of the largest pancreatic cancer GWAS datasets from the Pancreatic Cancer Cohort Consortium (PanScan) and the Pancreatic Cancer Case-Control Consortium (PanC4), encompassing 8,769 cases and 7,055 controls of EUR ancestry. The authors replicated six variants under a recessive inheritance model in 3,212 cases and 3,470 controls from the PANcreatic Disease ReseArch (PANDoRA) consortium (Campa et al., 2013), including 250 Brazilian cases and 70 controls. A meta-analysis confirmed that three SNPs (rs4626538, rs7008921, and rs147904962) showed recessive effects rather than additive effects, although none reached the conventional genome-wide threshold in the replication study. Additionally, rs36018702 was associated with *BCL2L11* and *BUB1* gene expression, implicating them in pancreatic tumor biology. Purdue et al. (2024) conducted a meta-analysis of multi-ancestry GWAS for Kidney Cancer (29,020 cases and 835,670 controls), including Brazilians (1,320 cases and 1,229 controls). Sixty-three susceptibility SNPs (50 novel) comprising 108 independent risk loci were identified, including a variant common among individuals of AFR descent (rs7629500). The PRS constructed from these loci demonstrated robust discriminatory performance in the EUR ancestry validation datasets. The Continental Origins and Genetic Epidemiology Network (COGENT)-Kidney Consortium was created to address the lack of ancestral diversity in kidney trait genomic studies by integrating non-EUR ancestry participants into multi-ancestry meta-analyses of estimated glomerular filtration rate (eGFR) (Franceschini and Morris, 2020). Building on this framework, Hughes et al. (2024) conducted a large GWAS meta-analysis of eGFR including 145,732 individuals with kidney cancer from cohorts across the Americas, comprising participants of AFR, NAM, and EUR ancestries. The analysis included two Brazilian cohorts, the Bambuí Brazil Cohort Study of Aging (BAMBUI) and the Brazilian Longitudinal Study of Adult Health (ELSA-Brasil). Forty-one significant loci were detected (two novel loci: *GABBR2* and *LCOR*). No allelic effect heterogeneity was observed according to ancestry or sex. Multi-ancestry PRS for eGFR showed increased, although variable, predictive power, underscoring the importance of population diversity in genomic research, including GWAS and multi-omics studies to enhance clinical translation for all ancestry groups.

### Metabolic diseases

*Overweight and obesity* are major drivers of metabolic disorders and risk factors for non-communicable diseases, accounting for 63% of deaths worldwide and 72% in Brazil (Schmidt et al., 2011). Body Mass Index (BMI) is a multifaceted polygenic trait shaped by interactions between environmental factors and thousands of genetic variants, in which each locus contributes only modestly to the overall phenotype (Robinson et al., 2017). The association between elevated BMI and Type 2 diabetes (T2D) is characterized by a complex interplay in which genetic predisposition to obesity serves as a precursor to metabolic dysfunction (Schmidt *et al.*, 2011). Scliar et al. (2021) performed admixture mapping and fine-mapping GWAS of BMI in three population-based Brazilian cohorts (a total of 8,965 participants) from the Northeast, South, and Southeast regions, representing three different life stages: childhood, young adulthood, and older adulthood. These cohorts were part of the EPIGEN-Brazil study (Kehdy et al., 2015). Six candidate FTO SNPs were associated with BMI in individuals of AFR or EUR ancestry. The analysis identified a West AFR-associated regulatory variant (rs114066381) with a sex-specific effect on BMI, which appears to play a significant role in the development of morbid obesity. Secolin et al. (2021b) inferred local ancestry for 125 exomes from Brazilian cohorts and compared them with two national genomic databases, BIPMed and ABraOM, as well as the 1000 Genomes Project Phase 3 and gnomAD datasets. The analysis included 954 Brazilian individuals (257 individuals from BIPMed, 609 from ABraOM, and 88 additional exomes from southeastern Brazil). The results support the hypothesis that variants located in the 8p23.1 region underwent positive selection during early admixture events. Sequencing of chromosome 8p23.1 identified 13 genes associated with obesity, T2D, lipid levels, and waist circumference (including *PRAG1*, *MFHAS1*, and *PPP1R3B*), which may influence obesity-related disease predisposition in admixed Brazilian populations.

*Glycemic traits* are central to the diagnosis and monitoring of T2D and cardiometabolic health. Based on this, Chen et al. (2021) aggregated GWAS data from up to 281,416 non-diabetic individuals (30% non-EUR ancestry), including a Brazilian cohort (1982 Pelotas Birth Cohort), and measured fasting glucose (FG), 2-hour post-challenge glucose, glycated hemoglobin (HbA1c), and fasting insulin levels. Trans-ancestry and single-ancestry meta-analyses identified 242 loci (99 novel), of which 80% showed no significant heterogeneity across the ancestries. This is the first study to assess the transferability of EUR-derived glycemic trait PRS to other ancestries and demonstrates markedly reduced predictive accuracy in non-EUR datasets. Notably, 24 of the 99 newly identified loci were discovered only after incorporating EAS, Hispanic (HISP), AFR-NAM, and South Asian (SAS) participants. These findings underscore the ancestry-driven differences in allele frequencies and effect sizes. Qu et al. (2023) developed an LD-pred-based, trans-ethnic obesity PRS through a collaboration involving six international research centers across the Americas (including 9,333 participants from Brazil), Europe, the Middle East, and Oceania, leveraging an international effort supported by the International Hundred K + Cohorts Consortium (IHCC). The PRS was based on GWAS meta-analysis data from 339,224 individuals of EUR ancestry, identifying 97 significant BMI-associated loci that together explained 2.7% of the BMI variation. The trans-ethnic PRS outperformed population-specific scores across all non-EUR ancestry populations and demonstrated predictive value across diverse ancestries, including EAS and admixed Brazilians. Downie et al. (2025) conducted a GWAS of BMI z-scores to identify genetic loci associated with early-childhood BMI across eight ancestrally diverse cohorts, including the 1982 Pelotas Birth Cohort from Brazil (2,705 participants). The results were combined via an inverse-variance-weighted fixed-effect meta-analysis with previously published GWAS summary statistics for childhood BMI from the Early Growth Genetics (EGG) Consortium and the Norwegian Mother and Child Cohort (MoBa), including a total of 84,804 individuals. Thirty-nine significant loci were identified (three novel: *EFNA5, DTWD2/RP11-2N5.1*, and *LSM14A*), each of which showed distinct associations with obesity-related traits. The direction of effect was generally consistent across populations, although some variability was observed in population-specific effects within the LAC cohorts. Carpena et al. (2025) explored the relationship between sleep-related PRS and accelerometer-based sleep metrics among 3,052 Brazilian adolescents, investigating potential mechanisms mediated by obesity and cortisol-related molecular pathways. Three sleep PRS were developed based on the most recent GWAS. The polygenic components identified in EUR adults partially explained the sleep-time window observed in Brazilian adolescents. BMI-related molecular pathways strengthened the association between sleep PRS and sleep duration, whereas cortisol pathway variants significantly influenced the genetic liability for sleep efficiency. 

### Neurological and psychiatric disorders

The human cortex governs complex cognitive and motor functions; however, its intricate structure is highly susceptible to a broad spectrum of neurodevelopmental and neurodegenerative disorders (Vuoksimaa et al., 2015). The genetic architecture of the cortex is highly polygenic, suggesting that distinct sets of genes are responsible for the development and organization of specific cortical regions (Grasby et al., 2020).

*Age-related cognitive decline* (ACD) is the progressive deterioration of cognitive function associated with aging. The contribution of genetic factors to individual susceptibility and the prevalence of this condition has received increasing attention in recent years (Lin et al., 2017). Gouveia et al. (2019) performed admixture mapping and fine-mapping GWAS to identify genetic factors associated with a 15-year cognitive trajectory in 1,407 older Brazilian adults (BAMBUI), comprising 14,956 Mini-Mental State Examination (MMSE) assessments. GWAS identified 24 associated SNPs, including a genomic region at 3p24.2, where increased NAM ancestry was significantly correlated with faster ACD, and an SNP (rs142380904) directly associated with ACD. 

Brain morphometry, assessed by magnetic resonance imaging (MRI), provides critical morphological markers of cortical structures. These measures are dynamic throughout life and fluctuate with age, serving as biological indicators of cognitive performance and susceptibility to psychiatric and neurodegenerative disorders (Lockhart and DeCarli, 2014). Hofer et al. (2020) conducted GWAS in large population-based samples from 20 discovery cohorts within the CHARGE consortium, including a Brazilian cohort (BRAINN-UNICAMP) and the UKBB, and replicated the strongest associations in 22,363 individuals from the Enhancing Neuroimaging Genetics through Meta-analysis (ENIGMA) consortium. Cortical measures across 34 predefined anatomical regions, including cortical thickness (CTh), cortical surface area (CSA), and cortical volume (CV), were analyzed in 22,824 participants using MRI. This study identified 160 significant loci linked to cortical structure, primarily involving the Wnt/β-catenin, transforming growth factor-beta (*TGF-β*), and sonic hedgehog signaling pathways. PRS analyses in 7,800 out-of-sample UKBB participants confirmed strong associations with all cortical measures. Genetic heterogeneity was observed across cortical traits and brain regions, with enrichment of genes related to anthropometric and vascular traits, hindbrain development, and neurodegenerative and psychiatric conditions. Grasby et al. (2020) conducted a meta-analysis of GWAS of brain MRI data from 51,665 individuals across 60 cohorts worldwide, including a Brazilian cohort (BRAINN-UNICAMP). The surface area (SA) and average thickness (TH) of the entire cortex and 34 functional regions were analyzed. A total of 199 significant loci were identified, with enrichment of loci influencing total SA within regulatory elements active during prenatal cortical development, supporting the radial-unit hypothesis. PRS analyses significantly predicted SA and TH in an independent sample of 5,095 EUR participants, explaining 23% of trait variance. Variations in cortical structure show significant genetic correlations with neurological and psychiatric disorders, confirming the highly polygenic architecture of cortical traits.

*Epilepsy* is a group of brain disorders characterized by recurrent, unprovoked seizures that affect up to 65 million individuals worldwide (Thurman et al., 2011). Genetic generalized epilepsy (GGE) is among the most common forms, with an estimated prevalence of 190 cases per 100,000 individuals (Aaberg et al., 2017). McCormack et al. (2018) examined genetic predictors of maculopapular exanthema (MPE), a cutaneous adverse reaction to antiepileptic drugs, in 323 cases and 1,321 drug-tolerant controls from epilepsy cohorts of northern EUR and ASI ancestry, including a replication cohort from Brazil. A GWAS included Class I and II human leukocyte antigen (HLA) alleles and identified an association between a rare variant in *CFHR4* and phenytoin-induced MPE in EUR individuals. This variant was in complete LD with a missense variant (N1050Y) in *CFH*, reinforcing the association between HLA-A*31:01 and carbamazepine hypersensitivity. Notably, a causal variant of *CFH* was found in a patient from Brazil. The International League Against Epilepsy (ILAE) Consortium on Complex Epilepsies, launched in 2011, reported its first meta-analysis in 2014, identifying loci at 2q24.3, 4p15.1, and 2p16.1 (Anney et al., 2014). Abou-Khalil et al. (2018) expanded this analysis to 15,212 individuals with epilepsy and 29,677 controls, including a Brazilian cohort (177 cases and 162 controls), and identified 16 significant loci (11 novel). Twenty-one candidate epilepsy genes were prioritized and were primarily linked to GGE. GWAS and exome sequencing have identified 48 SNPs associated with GGE. However, these studies were mainly based on non-admixed EUR and ASI populations (Abou-Khalil *et al.*, 2018). Thus, Kaibara et al. (2021) extracted genotypes for these 48 candidate SNPs from SNP-array data generated by the Genome-Wide Human SNP Array 6.0 for 87 Brazilian GGE patients and 340 controls in BIPMed and compared allele frequencies with those of the EUR, AFR, and NAM reference groups. Nine flanking SNPs across eight candidate loci were detected, replicating the previous findings. In addition, the two-proportion Z-test indicated that the lack of association among the remaining candidate SNPs may be due to differences in the genomic background observed among admixed Brazilians. This is the first study to analyze GGE-associated variants in an admixed Brazilian cohort, demonstrating the importance of considering population structure for accurate risk stratification. Stevelink et al. (2023) conducted a multi-ancestry GWAS including 29,944 cases (across three major categories and seven epilepsy subtypes) and 52,538 controls, including a Brazilian cohort (340 participants). Twenty-six significant loci were identified, of which 19 were specific to GGE, implicating 29 potentially causal genes. SNP-based heritability analyses indicated that common variants explain 39.6-90% of the genetic risk of GGE and its subtypes.

*Parkinson’s disease* (PD) is a progressive neurodegenerative disorder characterized by Lewy body pathology and loss of dopaminergic neurons in the midbrain. Identifying genetic risk factors is essential for mitigating the increasing global burden of PD (GBD 2016 Parkinson’s Disease Collaborators, 2018). The Latin American Research Consortium on the Genetics of PD (LARGE-PD) unites 35 institutions across 12 countries in the LAC region, including Brazilian cohorts (Zabetian and Mata, 2017). Loesch et al. (2021) performed the first GWAS of patients with PD in LAC populations, analyzing 1,497 participants (cases and controls) from the LARGE-PD consortium, including Uruguay, Peru, Chile, Colombia, and Brazil (148 cases and 227 controls). Over 8.7 million variants were genotyped using an array optimized for diverse ancestries (Multiethnic Genotyping Array [MEGA] from Illumina). Replication was conducted in 1,234 LAC PD cases and 439,522 controls from the 23andMe database. One significant locus, rs356182 (*SNCA*), was observed in both the discovery and replication phases of the study. Admixture mapping identified two significant loci: *STXBP6* in a NAM-ancestry joint test and *RPS6KA2* in an AFR-ancestry test, highlighting the ancestry-specific effects. Kim et al. (2024) performed a large-scale multi-ancestry and meta-analysis GWAS including 49,049 PD cases, 18,785 proxy cases (participants with a parent diagnosed with PD), and 2,458,063 controls from EUR, EAS, LAC (including Brazilian cohorts), and AFR ancestries. Seventy-eight significant loci were identified (12 novel loci: *MTF2, PIK3CA, ADD1, SYBU, IRS2, USP8, PIGL, FASN, MYLK2, USP25, EP300, and PPP6R2*). Six causal variants were fine mapped to known loci, advancing our understanding of PD risk in non-EUR populations.

*Attention-Deficit/Hyperactivity Disorder* (ADHD) affects 5-12% of children and adolescents and has a multifactorial etiology with high heritability (70-80%) (Franke et al., 2012). Rovira et al. (2020) conducted a meta-analysis of GWAS on persistent ADHD in adults and children, comparing genetic architectures across the lifespan in 17,149 cases and 32,411 controls, including two Brazilian cohorts and a Spanish cohort (10,617 cases and 16,537 controls). Nine novel loci were shared between childhood and adult ADHD (four loci, including genes involved in brain development and function: *FEZF1, RSPH3, CADPS2*, *AMN*, and *FBXL17*), supporting the hypothesis that persistent ADHD in adults is a neurodevelopmental disorder with high and significant genetic overlap with ADHD in children. The oxytocin-vasopressin (OT/AVP) pathway has emerged as a key neurobiological system implicated in ADHD (Le et al. 2021). Thus, Camerini et al. (2025) evaluated the association between a genome-wide ADHD PRS and an OT/AVP-specific PRS in participants from the 2004 Pelotas Birth Cohort, Brazil (N = 4,231). ADHD-PRS and OT/AVP-specific PRS were associated with ADHD symptoms, attentional control, selective attention, and verbal processing speed, supporting the role of the OT/AVP pathway in executive function and precision medicine approaches for ADHD.

*Machado-Joseph Disease* (MJD), the most common dominantly inherited ataxia worldwide, is an autosomal dominant neurodegenerative disorder characterized by progressive cerebellar ataxia and pyramidal signs (Bettencourt and Lima, 2011). An expanded CAG repeat in the *ATXN3* gene causes MJD, with repeat length partially explaining age-at-onset (AO) variability (de Mattos et al., 2019). Akçimen et al. (2020) conducted the first GWAS to identify potential genetic modifiers of AO in MJD, analyzing data from 786 patients from five regions, Portugal, North America, Germany, Australia, and Brazil (311 cases). Nine loci were identified that were enriched for genes involved in vesicle transport, olfactory signaling, and synaptic pathways. The associations between AO, *TRIM29,* and *RAG* suggest that DNA repair mechanisms contribute to MJD pathogenesis and highlight additional modifiers relevant to genotype-phenotype correlations.

*Schizophrenia* is a highly polygenic and heritable disorder (approximately 79-81% based on twin and nationwide population studies), emerging in late adolescence or early adulthood, and is influenced by numerous common alleles of small effect that are detectable through GWAS (Owen et al., 2016). Trubetskoy et al. (2022) conducted a two-stage GWAS including up to 76,755 schizophrenia cases and 243,649 controls from 90 Psychiatric Genomics Consortium (PGC) cohorts and incorporated nine AFR-NAM and LAC cohorts with Brazilian participants (the Federal University of São Paulo- UNIFESP Cohort). Common variant associations at 287 distinct loci were identified, concentrated in genes expressed in central nervous system neurons, both excitatory and inhibitory. Candidate genes (*GRIN2A* and *SP4*) were associated with rare disruptive coding variants of schizophrenia. Using all-ancestry primary GWAS as a discovery dataset, PRS analyses explained a median of 7.3% of the liability variance, reinforcing the highly polygenic nature of schizophrenia.

*Glaucoma* is increasingly recognized as a complex neurodegenerative disease and the leading cause of irreversible blindness. It is characterized by the progressive loss of retinal ganglion cell axons and associated visual field damage involving the central nervous system (CNS), which impacts cognitive function (Durán-Cristiano et al., 2025). Genetic studies have identified multiple loci linked to various forms of glaucoma, such as Primary Angle-Closure Glaucoma (PACG), whose epidemiological risk factors include advancing age, female sex, and EAS ancestry (Vithana et al., 2012). Khor et al. (2016) conducted a GWAS followed by replication in a combined cohort of 10,503 PACG cases and 29,567 controls from 24 countries across Asia, Australia, Europe, North America, and LAC (including Brazil with 103 cases and 203 controls). A meta-analysis identified significant associations with eight SNPs, encompassing five novel loci (*EPDR1*, *CHAT*, *GLIS3*, *FERMT2,* and *DPM2*-*FAM102A*) and three previously reported loci (*PLEKHA7*, *COL11A1*, and *PCMTD1-ST18*). These findings expand the genetic landscape of PACG and highlight shared and ancestry-specific risk factors for disease susceptibility across diverse populations.

### Coronavirus disease 2019

Coronavirus disease 2019 (COVID-19), caused by infection with severe acute respiratory syndrome coronavirus 2 (SARS-CoV-2), has resulted in a worldwide pandemic with enormous health and economic consequences (van der Made et al., 2020). Brazil has reported the third-highest number of COVID-19 infections worldwide (WHO, 2020), with the highest number of cases concentrated in the country’s southeastern region (Brasil, 2020b). Secolin et al. (2021 a ) analyzed 27 candidate genes and HLA alleles in 954 admixed Brazilian exomes using information from two public Brazilian databases (BIPMed and ABraOm) and additional exomes from individuals born in Southeast Brazil. Six variants previously reported to influence COVID-19 infection rates or clinical outcomes were detected. Seventy variants were identified exclusively in the Brazilian sample, of which seven were predicted to affect protein function (involving the loci *SLC6A20, LZTFL1, XCR1,* and *FURIN*). Furthermore, HLA alleles previously associated with the COVID-19 response at the *DQB1* and *DRB1* loci were identified. Niemi et al. (2021) reported the results of three GWAS meta-analyses comprising up to 49,562 patients with COVID-19 from 46 studies across 19 countries, including Brazil (2,245 cases and 2,819 controls). Thirteen significant loci associated with SARS-CoV-2 infection or severe manifestations of COVID-19 have been reported. Two loci that were associated with disease severity were identified only when the four studies of individuals with EAS ancestry were included. One of these loci, close to *FOXP4*, is common, particularly in EAS (32%), NAM (20%), and Middle Eastern (ME) (7%) ancestries, but has a low frequency in most EUR ancestries (2-3%). Pereira et al. (2022) conducted a GWAS for COVID-19 hospitalization, enrolling 3,533 cases and 1,700 controls in São Paulo (Brazil). A meta-analysis was also conducted, merging Brazilian Initiative on Precision Medicine and Personalized Health Care in COVID-19 (BRACOVID) hospitalization data with the Human Genetic Initiative (HGI) Consortium data. The BRACOVID results validated most loci previously identified in the HGI meta-analysis. Using only BRACOVID data, a new significant locus on Chr1 *(DSTYK* and *RBBP5*) was identified, which was specific to Brazilians. The associated haplotype has also been linked to several blood cell traits and may modulate the immune response in patients with COVID-19. Pairo-Castineira et al. (2023) analyzed 24,202 cases of COVID-19 with critical illness comprising a combination of microarray genotype and whole-genome sequencing data from cases of critical illness in the international Genetics of Mortality in Critical Care (GenOMICC) study, including the BRACOVID (5,223 Brazilians), combined with other studies recruiting hospitalized patients with a strong focus on severe and critical disease: the International Severe Acute Respiratory and Emerging Infections Consortium Coronavirus Clinical Characterization (ISARIC4C) and The Spanish Coalition to Unlock Research on Host Genetics on COVID-19 (SCOURGE) consortia. A total of 49 significant loci associations were identified (16 novel, including potentially druggable targets at *JAK1, PDE4A, SLC2A5, AK5, TMPRSS2,* and *RAB2A*). These data, together with ancestry-specific effects, may explain the heterogeneity observed in GWAS signals and deepen our understanding of the pathogenesis of critical COVID-19. Díaz-de Almeida *et al.* (2024) conducted a GWAS for COVID-19 hospitalization in admixed NAM populations, comprising 4,702 hospitalized cases recruited from the SCOURGE consortium and seven other participating studies in the COVID-19 HGI from LAC countries, including Mexico, Colombia, Paraguay, Ecuador, and Brazil (1,147 cases). Four significant associations were identified (two novel loci, which were first discovered in LAC populations: *BAZ2B* and *DDIAS*). A trans-ethnic meta-analysis revealed another novel cross-population risk locus in *CREBBP*. A cross-ancestry PRS in the SCOURGE-admixed NAM cohort effectively stratified individuals by genetic risk and was consistent with the patients’ clinical severity classification.

### Population stratification, methodological limitations, and challenges inherent to GWAS and PRS approaches

In admixed populations, local ancestry can facilitate the identification of causal variants that are often missed in EUR-centric studies. Brazilian genomic data illustrate this gap, as nearly half of the variants identified in the Brazilian populations are absent from global databases dominated by samples of EUR descent (Martin et al., 2017; da Cruz et al., 2022). Critical variants may therefore be overlooked, particularly when they are rare or population-specific, an issue that becomes increasingly relevant as the field shifts toward the study of rare variation influencing complex phenotypes (Wojcik et al., 2019).

The disease-centered approach adopted in this review enabled the evaluation of how genetic diversity and admixture patterns in Brazilian populations influence disease risk prediction and the transferability of genomic findings across ancestral backgrounds in different phenotypes (Table S1). A key limitation of GWAS and PRS studies in this context is the insufficient characterization of individual ancestry and its influence on genetic risk associations, particularly in multi-ancestry populations. This highlights the need to stratify admixed populations in case-control studies using genetically inferred ancestry groups, such as AFR, NAM, ASI, and EUR.

This review also revealed a marked geographic concentration of genomic research in Brazil, with most studies conducted in the Southeast and South regions, particularly in São Paulo (SP), Minas Gerais (MG), and Rio Grande do Sul (RS) (Figure 3). These regions receive disproportionately higher research funding, which is likely to contribute to their greater scientific output. However, this imbalance has important implications, as a substantial portion of Brazil’s genetic diversity is concentrated in populations from the North and Northeast, where NAM and AFR ancestries are more prevalent but remain underrepresented in genomic studies (Nunes et al., 2025).

Evidence from large multi-ancestry studies reinforces these observations. The Population Architecture using Genomics and Epidemiology (PAGE) study demonstrated that excluding non-EUR populations from genomic research contributes to disparities in precision medicine. By analyzing 26 phenotypes in nearly 50,000 individuals of diverse ancestries, the study identified marked allelic-effect heterogeneity across populations and showed that multi-ancestry cohorts improve fine-mapping resolution, which is essential for identifying causal variants (Wojcik et al., 2019).

A limitation of GWAS is its reliance on common variants, which often act as proxies for true causal mutations. These “tagging” SNPs may represent regulatory elements that influence gene expression independently of their genomic location or orientation (Khor et al., 2016). As a result, fine-mapping and functional studies are required to elucidate the biological mechanisms underlying GWAS associations, which remain a major challenge.

Furthermore, only nine of the 42 studies (21.4%) included in this review performed replication in independent cohorts (Table S1). Replication studies frequently report smaller effect sizes or fail to achieve statistical significance unless substantially larger sample sizes are used, which represents an additional limitation. Detecting variants with recessive effects is particularly challenging in small datasets unless they are either common or have large effect sizes (Lu et al., 2021). The relatively small sample sizes of Brazilian GWAS may therefore have limited the identification of additional genome-wide significant loci, particularly those with modest effects in admixed populations.

The clinical implementation of PRS is further constrained by reduced predictive accuracy in underrepresented admixed populations. In addition to ancestry-related biases in GWAS, PRS interpretability is influenced by sociodemographic factors and study design, which further limits clinical applicability (Mostafavi et al., 2020). In this review, 14 of the 42 studies (33%) included PRS analyses in Brazilian admixed populations. Furthermore, interpretation of meta-analyses also requires caution, given challenges related to case-control ascertainment and the potential for collider bias.

Finally, population biobanks, although usually providing larger datasets, often rely on self-reported clinical information or primary care codes, which may lack precise phenotype definitions and introduce phenotypic heterogeneity across studies. Thus, addressing these limitations will require more refined phenotyping strategies and better-curated datasets to improve the biological and clinical relevance of genomic findings.

### Challenges and opportunities for the implementation of genomics in medicine in Brazil

Global inequities continue to shape the integration of genomics into routine healthcare, with access to high-cost technologies largely concentrated in wealthier countries. Although declining sequencing costs are facilitating the expansion of genomics from monogenic to complex diseases, this transition may further exacerbate disparities in access to genomic testing and advanced therapies worldwide (Lopes-Cendes and de Oliveira, 2025).

Substantial data gaps persist for southern EUR (particularly Mediterranean), sub-Saharan AFR, and NAM populations, underscoring the need for research initiatives that better reflect global genetic diversity (Landry et al., 2018). In Brazil, the Unified Health System (SUS) has advanced genomic medicine through the 2014 Rare Diseases Policy and the 2020 incorporation of whole-exome sequencing. While these milestones represent important steps toward expanding access, achieving equitable implementation across regions remains a major challenge (Brasil, 2014, 2020c). Furthermore, large-scale initiatives generating high-coverage whole-genome sequencing data-such as BIPMed, Genomas Brasil, and DNABr-are beginning to address these gaps by building resources that capture Brazil’s genetic diversity. Collectively, these efforts are establishing the scientific and infrastructural basis for integrating genomics into routine medical care within the SUS.

At the global level, genomic equity has emerged as a central priority in the international health agenda. The World Health Organization (WHO) emphasized the importance of equitable access to genomics for achieving global health goals and established the Technical Advisory Group on Genomics (TAG-G), which brings together experts from diverse disciplines and regions (WHO, 2024). To improve the representation and transferability of GWAS and PRS findings across populations, several strategic actions have been proposed, including the development of regional genomic hubs to support data sovereignty, the strengthening of transnational collaborations across LAC, and the implementation of standardized ethical frameworks for responsible data sharing. Anchored in national capacity-building and regional cooperation, these strategies are essential to ensure that advances in genomics translate into equitable health benefits (Lopes-Cendes and de Oliveira, 2025).

## Conclusions

This review shows that GWAS and PRS analyses in Brazilian populations have advanced our understanding of how genetic variation contributes to complex diseases. Brazil’s distinctive three-way admixture of EUR, AFR, and NAM ancestries creates a genetic landscape that is not captured by studies restricted to predominantly EUR or ASI populations. Across multiple disease domains, ancestry-specific variants show distinct effect patterns, PRS developed in other populations display reduced predictive performance when applied to Brazilians, and novel associations have emerged that would likely remain undetected without research focused on this admixed population. Beyond identifying new loci, these studies have also provided insights into the biological basis of differential disease susceptibility. At the same time, the marked geographic concentration of genomic research within Brazil highlights an important limitation. Reducing this imbalance will be essential if genomic discovery and clinical translation are to reflect the full diversity of the Brazilian population.

Future success will depend on consolidating Brazil-specific genomic reference panels, fostering regional cooperation, and establishing centers of excellence in sequencing, bioinformatics, and clinical translation. These coordinated efforts are essential to translate genomic discoveries into tangible health benefits for Brazil’s diverse population. Ultimately, progress toward genomic equity will require not only technological advances and international collaboration, but also a sustained commitment to studying and incorporating the full spectrum of human genetic variation into research and clinical practice.

### Supplementary material

The following online material is available for this article:

Table S1 - List and summary information of the 42 GWA and PRS studies included in this systematic review.

## Data Availability

This is a systematic literature review, and no new data were generated.
