# Supplementary material for: Exploring genetic diversity in genome-wide association studies and polygenic risk scores in the admixed Brazilian population
Source: Genet Mol Biol. 2026 Aug 24;49(Suppl 1):e20250235. doi: 10.1590/1678-4685-GMB-2025-0235 (PMC13502495; doi:10.1590/1678-4685-GMB-2025-0235)
Supplement: Supplementary file 1 [file 1415-4757-GMB-49-s1-e20250235-s1.pdf]

Genetics and Molecular Biology
https://doi.org/10.1590/1678-4685-GMB-2025-0235

Supplementary Material to "Exploring genetic diversity in genome-wide association studies and polygenic risk scores in the admixed Brazilian population"

Table S1 - List and summary information of the 42 GWA and PRS studies included in this systematic review.

Reference	Phenotype	Study Type	Population/Ethnicity	Brazilian Cohort (Cases/Controls)	Origin of the Brazilian cohort (State)	Significant SNPs (N)	Key Findings
Christophersen *et al.*, 2017[1]	Atrial Fibrillation	Multi-ancestry, meta-analysis, case-control and replication GWAS	EUR, AFR-NAM, ASI, HISP, Brazilian	187/550	SP	12 (novel)	Identification of 12 AF loci including genes that encode transcription factors (*PITX2*, *ZFHX3*, *PRRX1*, *SOX5* and *TBX5*) that are involved in cardiac electrical and structural remodeling. A significant association of variants on 4q25 involving *PITX2* (rs6843082, OR 1.40, 95% CI 1.24–1.58, P=4.31×10^{-8}) between AFR-NAM and ASI ancestry (rs2723334, OR 1.94, 95% CI 1.68–2.25, P=8.46×10^{-19}) and EUR ancestry (rs2129977, OR 1.45, 95% CI 1.41–1.49, P=7.25×10^{-136}) was observed with AF.
Roselli *et al.*, 2018[2]	Atrial Fibrillation	Multi-ancestry and meta-analyses GWAS	EUR, ASI, AFR-NAM, HISP, Brazilian	568/1,096	SP	97 (67 novel)	Association of 97 loci with AF, 67 of which were novel in a combined-ancestry analysis and three in a EUR specific analysis. Genes at AF-associated loci were identified in 101 left atrial samples through RNA sequencing and eQTL analyses. 57 AF-associated genes, 42 of which overlap with GWAS loci was identified by transcriptome-wide analyses.
Ntalla *et al.*, 2020[3]	Cardiac Conduction (PR interval)	Multi-ancestry and meta-analyses GWAS & PRS	EUR, AFR, HISP, Brazilian	485 cases	MG	202 (141 novel)	Report signals in 12 candidate genes at previously not reported loci with functional roles in cytoskeletal assembly (*DSP, DES, OBSL1,*

Reference	Phenotype	Study Type	Population/Ethnicity	Brazilian Cohort (Cases/Controls)	Origin of the Brazilian cohort (State)	Significant SNPs (N)	Key Findings
							PDLIM5, LDB3, FHL2, CEFIP, SSPN, TLN2, PTK2, GJA5, and *CDH2*). The PR interval analysis could identify distinct pathophysiologic mechanisms leading to AF and genetic relationship between PR interval duration and arrhythmias.
Thériault *et al.*, 2022[4]	Premature Atrial Contractions & Atrial Fibrillation	Meta-analysis and replication GWAS	EUR	405 cases (BHS cohort)	SP	6	A novel association at the *SCN5A* locus (including the SNP rs7373862) with PAC and AF frequency, that was associated with an increase of 0.12 [95% CI 0.08–0.16] standard deviations of the normalized PAC frequency per risk allele.
Young *et al.*, 2023[5]	Cardiovascular Disease/ Cardiac Electrical Biomarkers	Multi-ancestry and meta-analyses GWAS & PRS	EUR, HISP/LA, AFR, EAS, SAS	518 cases (BAMBUI cohort)	MG	61 (58 novel)	Sixty-one loci (58 novel) associated with the spatial QRS-Ta (sQRS-Ta; N= 118,780) and 11 loci associated with the frontal QRS-Ta (fQRS-Ta; N= 159,715) were identified; implicating candidate genes involved in cardiovascular diseases (*MYH7, TTN, TNNT2, MYBPC3, DSP* and *RBM20*).
Carneiro-Proietti *et al.*, 2018[6]	Sickle Cell Disease	Characterization GWAS	Brazilian	2,795 cases	MG, RJ, PE, SP	-	HbSS was the most common SCD genotype (70.7%), followed by HbSC (23%), Sβ0 (3.0%) and Sβ+ (2.9%). SNP data from the TM Array were analyzed to evaluate the genetic ancestry of the cohort and reveal significant admixture among the population.
Ibanez *et al.*, 2022[7]	Acute Ischemic Stroke (outcome)	Multi-ancestry and meta-analysis GWAS	Mixed ancestry cohorts (non-Hispanic white, Hispanic, Korean and AFR-NAM), including Brazilian (MEGASTROKE study)	NA (BRAINN/ UNICAMP cohort)	SP	8	Detection of eight associated loci (1p21.1, 1q42.2, 2p25.1, 2q31.2, 2q33.3, 5q33.2, 7p21.2 and 13q31.1); and association of *ADAM23* (2q33.3); *GRIA1* (5q33.2); and *GNPAT* (1p21.1) and *ABCB5* (7p21.1) genes with modulation of neuronal excitability contributing to

Reference	Phenotype	Study Type	Population/Ethnicity	Brazilian Cohort (Cases/Controls)	Origin of the Brazilian cohort (State)	Significant SNPs (N)	Key Findings
							early neurological instability after AIS by expression quantitative trait loci mapping and summary data-based Mendelian Randomization. RNA-sequencing indicates that the gene expression of *ADAM23* and *GRIA1* are enriched in neurons and made part of a synaptic protein complex that modulates neuronal excitability.
Mishra *et al.*, 2022[8]	Stroke	Cross-ancestry, meta-analysis and case-control GWAS & PRS	EUR, EAS, AFR-NAM, SAS, HISP	NA (Joinville Biobank-cases/controls)	SC	89 (61 novel)	Implication of putative causal genes (*SH3PXD2A* and *FURIN*) and variants (*GRK5* and *NOS3*) for stroke risk by cross-ancestry fine-mapping, in silico mutagenesis analysis, and transcriptome-wide and proteome-wide association analyses. Using a three-pronged approach, genetic evidence provided for putative drug effects (*F11*, *KLKB1, PROC, GP1BA, LAMC2* and *VCAM1*) as possible targets. PRS integrating cross ancestry-specific stroke GWAS with vascular risk factor strongly predicted IS risk (~2-2.5-fold) for EUR and EAS populations, and 1.5-fold for AFR ancestry.
Earley *et al.*, 2023[9]	Sickle Cell Disease/ Early Ischemic Stroke	Case-control GWAS	Brazilian	178/1,155	MG, RJ, PE, SP	14	Identification of previously non-SCD EIS genes (<65 years): *ADAMTS2* ($p = 3.70 \times 10^{-9}$) and *CDK18* ($p = 2.38 \times 10^{-9}$); and 14 novel genes ($p < 5 \times 10^{-8}$). Meta- analysis, showed association for variants in independent SCD cohorts: rs1209987- *TBC1D32* ($p = 3.36 \times 10^{-10}$), rs188599171- *CUX1* ($p = 5.89 \times 10^{-11}$), rs77900855- *BTG1* ($p = 4.66 \times 10^{-8}$), and rs141674494- *VPS13C* (1.68×10^{-9}).

Reference	Phenotype	Study Type	Population/Ethnicity	Brazilian Cohort (Cases/Controls)	Origin of the Brazilian cohort (State)	Significant SNPs (N)	Key Findings
Savage *et al.*, 2013[10]	Osteosarcoma (susceptibility)	Multi-stage and meta-analysis GWAS	EUR	247 cases (GRAAC/UNIFESP-replication stage)	SP	3	Implication of three susceptibility loci: *GRM4* gene (rs1906953 at 6p21.3, $P = 8.1 \times 10^{-9}$) and a gene desert (rs7591996 and rs10208273 at 2p25.2, $P = 1.0 \times 10^{-8}$ and 2.9×10^{-7}) on the biological mechanisms of osteosarcoma.
Mirabello *et al.*, 2015[11]	Osteosarcoma (metastasis)	Multi-stage, meta-analysis and replication GWAS	EUR, AFR, Brazilian	107 cases	SP	1	Association of the *NFIB* gene (rs7034162 at 9p24.1) with metastasis osteosarcoma cases with EUR, AFR and Brazilian ancestry (meta-analysis of all cases: $P = 1.2 \times 10^{-9}$, OR 2.43, 95% CI 1.83–3.24). A transposon screen in mice identified that a significant proportion of osteosarcomas harboring inactivating insertions in *Nfib* had lowered Nfib expression.
Lesseur *et al.*, 2016[12]	Oral Cavity and Oropharyngeal Cancer	Meta-analysis GWAS	12 studies from Europe, North America, and South America (including Brazil)	529/535 (GENCAPO), 457/600 (IARC- LA)	RJ, RS, SP	8 (7 novel)	Association of three loci at 6p21.32 (rs3828805, *HLA-DQB1*), 10q26.13 (rs201982221, *LHPP*), and 11p15.4 (rs1453414, *OR52N2/TRIM5*) with OC and OPC cancer combined; and four loci at 2p23.3 (rs6547741, *GPN1*), 9q34.12 (rs928674, *LAMC3*), 9p21.3 (rs8181047, *CDKN2B-AS1*) and 5p15.33 (rs10462706, *CLPTM1L*) with OC cancer.
Mathias *et al.*, 2023[13]	Breast Cancer	Case-control GWAS	Brazilian	291/370	PR	4	The SNPs rs4415084 and rs7716600 were associated with BC development at higher risk and with progesterone status and lymph node status, respectively. The rs3803662/rs4784227 haplotype GT was associated with BC risk.
Barreiro *et al.*, 2024[14]	Breast Cancer	Case-control GWAS & PRS	Brazilian (EUR, AFR, EAS)	1,606 cases (753-SABE and 853-BRGP, nationwide sample)	SP	313	Distribution of the 313-PRS is inflated when compared to that of the UKBB, leading to potential overestimation of PRS-based risk if

Reference	Phenotype	Study Type	Population/Ethnicity	Brazilian Cohort (Cases/Controls)	Origin of the Brazilian cohort (State)	Significant SNPs (N)	Key Findings
							the EUR component is taken as a standard. Case-controls lead to equivalent predictive power when compared to UKBB-EUR samples with AUROC values of 0.66-0.62 compared to 0.63 for UKBB.
Lu *et al.*, 2021[15]	Pancreatic Cancer	Meta-analysis and replication GWAS	EUR and Brazilian	250/70	PR, SP	6	Three SNPs: rs4626538 (7q32.2), rs7008921 (8p23.2) and rs147904962 (17q21.31) showed specific recessive effects, although none of the six SNPs reached the conventional threshold for genome-wide significance in the replication study. Indication of a relationship between rs36018702 and expression of the *BCL2L11* and *BUB1* genes involved in Pancreatic Cancer biology.
Purdue *et al.*, 2024[16]	Kidney Cancer	Multi-ancestry and meta-analysis GWAS & PRS	EUR, AFR, EAS, LA	1,320/1,229	SP	63 (50 novel)	Identification of 63 susceptibility loci of KC, including rs7629500, in the 3′ untranslated region of *VHLa*, common in AFR ancestry; and candidate functional variants localizing to eQTLs (*IRF5)* and TF-binding sites (*HIF*). PRS constructed from identified KC loci demonstrated good discriminatory performance in our validation dataset (AUC of 0.74 X 0.71 with risk factors only).
Hughes *et al.*, 2024[17]	Kidney function/ Kidney traits	Multi-ancestry and meta-analysis GWAS	AFR, NAM, HISP, LA	NA (BAMBUI and ELSA Cohorts)	BA, ES, MG, RJ, RS, SP	41 (2 novel)	Identification of 41 associated SNPs with KC ($p < 5 \times 10^{-8}$). Meta-analysis of GWASs of AFR and NAM ancestries identifies two new loci for *eGFR* (near *GABBR2*, rs73490762, $p = 6.3 \times 10^{-9}$ and *LCOR*, rs12258469, $p = 3.5 \times 10^{-8}$). PRS performance into AFR/AMR studies depends on ancestry and sample size.

Reference	Phenotype	Study Type	Population/Ethnicity	Brazilian Cohort (Cases/Controls)	Origin of the Brazilian cohort (State)	Significant SNPs (N)	Key Findings
Scliar *et al.*, 2021[18]	Body Mass Index	Admixture mapping and replication GWAS	Brazilian	8,965 cases (part of EPIGEN initiative)	BA, MG, RS	6	Identification of six SNPs (rs9930333, rs62033400, rs8050136, rs3751812, rs1558902 and rs9939609) associated with AFR or EUR ancestry and BMI; and the West-AFR variant (rs114066381) association with a female-specific effect on BMI.
Secolin *et al.*, 2021b[19]	Overweight/Obesity/Type 2 Diabetes	Multi-ancestry, meta-analysis exome and replication GWAS	Brazilian	954 cases (BIPMED, ABraOM, 1000 Genomes Project Phase 3 and gnomAD datasets)	MG, SP	13	Implication of 13 variants located at 8p23.1 (*PRAG1, MFHAS1, PPP1R3B, TNKS, MSRA, PRSS55, RP1L1, PINX1, MTMR9, FAM167A, BLK, GATA4*, and *CTSB*), which may influence obesity-related disease predisposition in admixed individuals of the Brazilian population.
Chen *et al.*, 2021[20]	Glycemic traits /Type 2 Diabetes	Trans-ancestry and meta-analysis GWAS & PRS	Global multi-ancestry (EUR, EAS, HISP, AFR-NAM, SAS, and sub-Saharan AFR)	NA (1982 Pelotas Birth Cohort)	RS	242 (99 novel)	Identification of 242 loci (99 novel; $P<5x10^{-8}$). Analyses restricted to EUR ancestry would have led to 24 fewer new loci. Each trait-specific PRS improves trait variance explained by between 3.5-fold (*HbA1c*) and 6-fold (*FG*) in the EUR dataset compared to a score built only from trans-ancestry lead variants and EUR index variants.
Qu *et al.*, 2023[21]	Body Mass Index	Trans-ethnic and meta-analysis GWAS & PRS	EUR, AFR-NAM, HISP/LA, EAS/SAS	9,333 cases (ELSA-Brazil cohorts)	BA, ES, RJ, RS, SP	97	The trans-ethnic PRS model tested in house showed AUC > 0.720 to predict the top 1% BMI was achieved in all the PRS models with mixture probability ≥ 0.03. The highest AUC is seen at the mixture probabilities of 0.03-0.1. The trans-ethnic PRS models outperformed the ancestry specific models in the non-EUR populations tested including EAS and Brazilian ancestries.
Downie *et al.*, 2025[22]	Childhood BMI	Trans-ancestry GWAS	Multiple cohorts (including Brazil)	2,705 cases (1982 Pelotas Birth Cohort)	RS	39 (3 novel)	Identification of 39 loci associated with childhood BMI, including three

Reference	Phenotype	Study Type	Population/Ethnicity	Brazilian Cohort (Cases/Controls)	Origin of the Brazilian cohort (State)	Significant SNPs (N)	Key Findings
							putatively novel loci (*EFNA5* and *DTWD2*, *RP11-2N5.1* on chr 5, and *LSM14A* on chr 19) with distinct effects across obesity-related traits in childhood and adulthood.
Carpena *et al.*, 2025[23]	Obesity & Stress/ Sleep metrics	GWAS & PRS	Brazilian	3,052 cases (1982 Pelotas Birth Cohort)	RS	941 BMI SNPs and 20 cortisol SNPs	The N. of nocturnal sleep episodes-PRS was positively associated with sleep time window ($p = .011$); with sleep time window when restricted to BMI-PRS ($p = .003$); and both the number of sleep episodes and sleep time window cortisol-PRS were associated ($p = .013$; $p = .003$, respectively) with sleep efficiency. Sleep polygenetic components observed in EUR adults may partially explain the accelerometer-based sleep time window in Brazilian adolescents.
Gouveia *et al.*, 2019[24]	Age-related Cognitive Decline	Admixture and fine-mapping GWAS	Brazilian- AFR, EUR, NAM	1,407 cases (BAMBUI cohort)	MG	24	Identification of a genomic region (3p24.2) in which increased NAM ancestry was significantly associated with faster ACD; and a SNP rs142380904 ($p = 7.5 \times 10^{-5}$) associated with ACD. Top six SNPs (rs138347004, rs76238412, rs142380904, rs190722109, rs78400729, and rs77499314) accounted for 18.5% of the ACD variance.
Hofer *et al.*, 2020[25]	Cortical structure; cognitive/Psychiatric traits (PD, insomnia, depression, neuroticism, ADHD)	Meta-analysis and replication GWAS & PRS	20 CHARGE cohorts (including Brazil) & UKBB	NA (BRAINN/UNICAMP cohort)	SP	160	Identification of 160 genome-wide significant associations pointing to wnt/β-catenin, *TGF-β* and sonic hedgehog pathways. PRS showed associations (pPRS $<4.76 \times 10^{-3}$) with all investigated cortical measures in all cortical regions.
Grasby *et al.*, 2020[26]	Cortical surface area/ thickness; Cognitive and Psychiatric traits	Meta-analysis and replication GWAS & PRS	60 Global cohorts (including Brazil)	NA (BRAINN/ UNICAMP cohort)	SP	199	Identification of 199 significant loci ($P < 8.3 \times 10^{-10}$; 187 influencing SA and 12 influencing thickness); and

Reference	Phenotype	Study Type	Population/Ethnicity	Brazilian Cohort (Cases/Controls)	Origin of the Brazilian cohort (State)	Significant SNPs (N)	Key Findings
							loci that affect regional SA clusters near genes in *Wnt* signaling pathways. PRS significantly predicted SA and TH in an independent sample explaining between 2-3% of the trait variance (P ≤ 0.01 R2 SA = 0.029).
McCormack *et al.*, 2018[27]	Adverse drug reaction in Epilepsy treatment	Meta-analysis and case-control and replication GWAS	EUR, ASI, including Brazil	NA (Replication sites cohorts in Brazil)	SP	1	Association between a rare variant (rs78239784) in the *CFHR4* gene and phenytoin-induced MPE in EUR; and between HLA-A*31:01 and carbamazepine hypersensitivity.
Abou-Khalil *et al.*, 2018 (ILAE)[28]	Epilepsy	Mega-analysis GWAS	Caucasian, ASI, AFR-NAM	177/162	SP	16 (11 novel)	Association of 21 genes with Epilepsy (the majority of these in GGE), including coding for ion-channel subunits, transcription factors and a vitamin-B6 metabolism enzyme.
Kaibara *et al.*, 2021[29]	Genetic Generalized Epilepsy	Case-control GWAS	Brazilian	87/340	SP	8	Replication of the association signals in eight candidate regions (rs12726617; rs9428842; rs1915992; rs1464634; rs6459526; rs2510087; rs9551042; rs9888879; and rs8133217, p-values $<3.55e^{-06}$) improving the risk stratification estimation in admixed individuals with GGE.
Stevelink *et al.*, 2023 (ILAE)[30]	Epilepsy	Multi-ancestry and meta-analysis GWAS	EUR, AFR, ASI	340 cases/controls (not specified)	SP	26 (19 GGE-specific)	Implication of 29 likely causal epilepsy genes (the majority of these overlapping with monogenic epilepsy genes and targets of current antiseizure medications). SNP-based heritability analyses indicated that common variants explain 39.6-90% of genetic risk for GGE and its subtypes
Loesch *et al.*, 2021[31]	Parkinson's Disease	Admixture fine-mapping and replication GWAS	9 LARGE-PD cohorts in Uruguay, Peru, Chile, Colombia, Brazil	148/227	RS, SP	6 (not replicated)	Association of the PD locus: *SNCA* (p-value $<5\ 10^{-8}$); rs356182 in both the discovery and the replication cohorts (discovery, G allele: 1.58

Reference	Phenotype	Study Type	Population/Ethnicity	Brazilian Cohort (Cases/Controls)	Origin of the Brazilian cohort (State)	Significant SNPs (N)	Key Findings
							OR, 95% CI 1.35–1.86, p-value $2.48x10^{-8}$; 23andMe, G allele: 1.26 OR, 95% CI 1.16–1.37, p-value $4.55x10^{-8}$); *STXBP6* (chr 14) in a joint test of ancestries and NAM single-ancestry test (p-value <$5\ 10^{-5}$); and *RPS6KA2* (chr 6) in the AFR single-ancestry test (p-value <5×10^{-5}).
Kim *et al.*, 2024[32]	Parkinson's Disease	Multi-ancestry and meta-analysis GWAS	EUR, EAS, LA and AFR	NA	MG, RS, SP	78 (12 novel)	Identification of 12 novel PD loci (*MTF2, PIK3CA, ADD1, SYBU, IRS2, USP8, PIGL, FASN, MYLK2, USP25, EP300* and PPP6R2) and six putative causal variants at six known PD loci (*TMEM163, TMEM175, SNCA, CAMK2D, HIP1R* and *LSM7*).
Rovira *et al.*, 2020[33]	Attention Deficit Hyperactivity Disorder	Meta-analysis GWAS & PRS	19 GWAS from PGC, iPSYCH, IMpACT, including 2 Brazilians cohorts	10,617/16,537 (comprising two brazilians cohorts and a Spanish cohort)	RS	13 (9 novel)	Identification of four new loci including genes involved in brain formation and function (*FEZF1, RSPH3, CADPS2, AMN* and *FBXL17*) associated with ADHD. PRS analyses showed that childhood ADHD-PRS were associated with persistent ADHD at different predefined P value thresholds, with the P = 0.40 threshold (N. SNPs =20,398) explaining the most variance (r2 = 0.0041 and P = 1.20E-27).
Camerini *et al.*, 2025[34]	Attention Deficit Hyperactivity Disorder	Meta-analysis GWAS & PRS	Brazilian	4,231 cases (2004 Pelotas Birth Cohort)	RS	27 (discovery study)	ADHD-PRS was associated with ADHD symptoms and attentional control/switching domain in children with ADHD. OT/AVP_{ADH}-PRS showed an enrichment for selective attention domain (P = 0.025); attention score (P = 0.050), and in attentional control/switching domain [verbal processing speed (P = 0.041); and attentional control (P = 0.033)].

Reference	Phenotype	Study Type	Population/Ethnicity	Brazilian Cohort (Cases/Controls)	Origin of the Brazilian cohort (State)	Significant SNPs (N)	Key Findings
Akçimen *et al.*, 2020[35]	Machado-Joseph disease	Multi-ancestry and meta-analysis GWAS	Five cohorts: Portugal, Brazil, North America, Germany and Australia	311 cases	RS, SP	9	Identification of nine genetic loci ($P < 1 \times 10^{-5}$) that may modify the age onset of MJD. The *TRIM29* and *RAG* were implicated on DNA repair, olfactory signaling, synaptic, and vesicle transport pathways in the pathogenesis of MJD.
Trubetskoy *et al.*, 2022[36]	Schizophrenia	Meta-analysis GWAS & PRS	90 PGC cohorts (EUR, EAS) & 9 AFR-NAM cohorts & LA cohorts (including Brazil)	NA (UNIFESP Cohort)	SP	287	Identification of 120 genes, including 16 with credible causal non-synonymous or UTR variation. Candidate genes (*GRIN2A* and *SP4*) were associated with rare disruptive coding variants in schizophrenia. PRS analysis explained a median of 0.073 of variance in liability (SNPs with GWAS $p<0.05$), and 0.024 when restricted to genome-wide significant SNPs.
Khor *et al.*, 2016[37]	Primary Angle Closure Glaucoma	Discovery and meta-analysis GWAS	24 countries across Asia, Australia, Europe, North America, and South America (including Brazil)	103/203	SP	8 (5 novel)	Association of five new PACG genetic loci: *EPDR1* (rs3816415), *CHAT* (rs1258267), *GLIS3* (rs736893) *FERMT2* (rs7494379) and *DPM2-FAM102A* (rs3739821); and three previously described loci at *PLEKHA7, COL11A1*, and *PCMTD1-ST18.*
Secolin *et al.*, 2021a[38]	COVID-19 (host genetics in Brazilians)	Exome GWAS	Brazilian	954	MG, SP	70 (Brazil-specific)	Detection of seven exclusively Brazilians variants at the loci (*SLC6A20, LZTFL1*, *XCR1* and *FURIN*) predicted to affect protein function and may be involved in COVID-19. In addition, HLA alleles (*DQB1* and *DRB1*), previously reported, were associated with the COVID-19 response.
Niemi *et al.*, 2021 (COVID-19 HGI)[39]	COVID-19	Meta-analysis GWAS	46 studies across 19 countries, including Brazil	2,245/2,819 (BRACOVID study)	SP	13	Implication of nine loci with biologically plausible genes associated with COVID19 ($P < 0.004$), including two locus affecting disease severity only in individuals

Reference	Phenotype	Study Type	Population/Ethnicity	Brazilian Cohort (Cases/Controls)	Origin of the Brazilian cohort (State)	Significant SNPs (N)	Key Findings
							with EAS ancestry, and one locus (*FOXP4*) commonly in EAS, NAM, ME and EUR ancestries.
Pereira *et al.*, 2022[40]	COVID-19 (severity)	Meta-analysis GWAS	Brazilian	3,533/1,700	SP	5 (1 novel, specific Brazil)	Association of one SNP (5×10^{-8}) at Chr 1 nearby *DSTYK* and *RBBP5* genes, which may play a role in modulating the immune response in COVID-19.
Pairo-Castineira *et al.*, 2023[41]	COVID-19 (critical illness)	Trans-ancestry and meta-analyses GenOMICC GWAS	GenOMICC and ISARIC4C studies & SCOURGE consortium	5,223 cases (BRACOVID study)	SP	49 (16 novel)	Identification of 16 novel variants associated with COVID-19, including potentially druggable targets in *JAK1, PDE4A, SLC2A5, AK5* genes and host factors required for viral entry and replication (*TMPRSS2* and *RAB2A*).
Diz-de Almeida *et al.*, 2024[42]	COVID-19 (hospitalization risk)	Trans-ethnic and meta-analysis GWAS & PRS	SCOURGE LA cohort & COVID-19 HGI studies	1,147 cases	DF, PA, PB, PE, RN	4 (2 novel)	Identification of two novel loci (*BAZ2B* and *DDIAS*) associated with COVID-19 in LA populations; and another novel cross-population risk locus in *CREBBP*. The PRS model was associated with a 1.48-fold increase in COVID-19 hospitalization risk per every PRS standard deviation.

Abbreviations: ACD: Age-related cognitive decline; ADHD: Attention Deficit Hyperactivity Disorder; AF: Atrial fibrillation; AFR: African; AIS: Acute Ischemic Stroke; ASI: Asian; AUC: Area Under the Curve; AUROC: Area Under the Receiver Operating Characteristic Curve; BA: Bahia; BAMBUI: Bambui Brazil Cohort Study; BC: Breast Cancer; BHS: Baependi Heart Study; BMI: Body Mass Index; CI: Confidence Interval; DF: Distrito Federal; EAS: East Asian; eGFR: estimated Glomerular Filtration Rate; EIS: Early Ischemic Stroke; ELSA-Brazil: Brazilian Longitudinal Study of Adult Health; eQTL: expression Quantitative Trait Locus; ES: Espírito Santo; EUR: European ; fQRS-Ta: frontal QRS-Ta,; spatial QRS-Ta; GGE: Genetic Generalized Epilepsy; GWAS: Genome-wide Association Study; HbSS/HbSC: Hemoglobin genotypes (SS and SC); HISP: Hispanic; HLA: Human Leukocyte Antigen; IS: Ischemic Stroke; KC: Kidney Cancer; LA: Latin American; ME: Middle Eastern; MG: Minas Gerais; MJD: Machado-Joseph Disease; N: Number; NA: Not available; NAM: Native American; OC/OPC: Oral Cavity/Oropharyngeal Cancer; OR: Odds Ratio; OT/AVP: oxytocin-vasopressin; PA: Pará; PAC: Premature Atrial Contraction; PACG: Primary Angle Closure Glaucoma; PB: Paraíba; PD: Parkinson' Disease; PE: Pernambuco; PR: Paraná; PR interval: P wave and R wave interval (electrocardiographic marker); PRS: Polygenic Risk Score; QRS-Ta: QRS complex and T-wave angle (electrocardiographic marker); RJ: Rio de Janeiro; RN: Rio Grande do Norte; RS: Rio Grande do Sul; SA: Surface Area; sQRS-Ta: spatial QRS-T angle; TH: Thickness; SABE: Health, Welfare and Aging Project; SAS: South Asian; SC: Santa Catarina; SCD: Sickle Cell Disease; SD: Standard Deviation; SNPs: Single Nucleotide Polymorphisms; SP: São Paulo; UKBB: UK Biobank.

References

1. Christophersen IE, Rienstra M, Roselli C, Yin X, Geelhoed B, Barnard J, Lin H, Arking DE, Smith AV, Albert CM *et al.* (2017) Large-scale analyses of common and rare variants identify 12 new loci associated with atrial fibrillation. Nat Genet 49:946–952.
2. Roselli C, Chaffin MD, Weng L-C, Aeschbacher S, Ahlberg G, Albert CM, Almgren P, Alonso A, Anderson CD, Aragam KG *et al.* (2018) Multiethnic genome-wide association study for atrial fibrillation. Nat Genet 50:1225–1233.
3. Ntalla I, Weng LC, Cartwright JH, Hall AW, Sveinbjornsson G, Tucker NR, Choi SH, Chaffin MD, Roselli C, Barnes MR *et al.* (2020) Multi-ancestry GWAS of the electrocardiographic PR interval identifies 202 loci underlying cardiac conduction. Nat Commun 11:2542.
4. Thériault S, Imboden M, Biggs ML, Austin TR, Aeschbacher S, Schaffner E, Brody JA, Bartz TM, Risch M, Grossmann K *et al.* (2022) Genome-wide analyses identify SCN5A as a susceptibility locus for premature atrial contraction frequency. iScience 25:105210.
5. Young WJ, Haessler J, Benjamins JW, Repetto L, Yao J, Isaacs A, Harper AR, Ramirez J, Garnier S, van Duijvenboden S *et al.* (2023) Genetic architecture of spatial electrical biomarkers for cardiac arrhythmia and relationship with cardiovascular disease. Nat Commun 14:1411.
6. Carneiro-Proietti ABF, Kelly S, Miranda Teixeira C, Sabino EC, Alencar CS, Capuani L, Salomon Silva TP, Araujo A, Loureiro P, Máximo C *et al.* (2018) Clinical and genetic ancestry profile of a large multi-centre sickle cell disease cohort in Brazil. Br J Haematol 182:895–908.
7. Ibanez L, Heitsch L, Carrera C, Farias FHG, Del Aguila JL, Dhar R, Budde J, Bergmann K, Bradley J, Harari O *et al.* (2022) Multi-ancestry GWAS reveals excitotoxicity associated with outcome after ischaemic stroke. Brain 145:2394–2406.
8. Mishra A, Malik R, Hachiya T, Jürgenson T, Namba S, Posner DC, Kamanu FK, Koido M, Le Grand Q, Shi M *et al.* (2022) Stroke genetics informs drug discovery and risk prediction across ancestries. Nature 611:115–123.
9. Earley EJ, Kelly S, Fang F, Alencar CS, Rodrigues D de OW, Soares Cruz DT, Flanagan JM, Ware RE, Zhang X, Gordeuk V *et al.* (2023) Genome-wide association study of early ischaemic stroke risk in Brazilian individuals with sickle cell disease implicates ADAMTS2 and CDK18 and uncovers novel loci. Br J Haematol 201:343–352.
10. Savage SA, Mirabello L, Wang Z, Gastier-Foster JM, Gorlick R, Khanna C, Flanagan AM, Tirabosco R, Andrulis IL, Wunder JS *et al.* (2013) Genome-wide association study identifies two susceptibility loci for osteosarcoma. Nat Genet 45:799–803.
11. Mirabello L, Koster R, Moriarity BS, Spector LG, Meltzer PS, Gary J, Machiela MJ, Pankratz N, Panagiotou OA, Largaespada D *et al.* (2015) A Genome-wide scan identifies variants in NFIB associated with metastasis in patients with osteosarcoma. Cancer Discov 5:920–931.
12. Lesseur C, Diergaarde B, Olshan AF, Wünsch-Filho V, Ness AR, Liu G, Lacko M, Eluf-Neto J, Franceschi S, Lagiou P *et al.* (2016) Genome-wide association analyses identify new susceptibility loci for oral cavity and pharyngeal cancer. Nat Genet 48:1544–1550.
13. Mathias C, Marin AM, Kohler AF, Sanchuki HBS, Sukow N, Beltrame MH, Baal SCS, Sebastião APM, de Souza Fonseca Ribeiro EM, Gradia DF *et al.* (2023) LncRNA-SNPs in a Brazilian breast cancer cohort: A case-control study. Genes (Basel) 14:971.
14. Barreiro RAS, Almeida TF, Gomes CS, Monfardini F, Farias AA, Tunes GC, Souza GM, Duim E, Correia JS, Coelho AVC *et al.* (2024) Assessing the risk stratification of breast cancer polygenic risk scores in a Brazilian cohort. J Mol Diagn 26:825–831.
15. Lu Y, Gentiluomo M, Macauda A, Gioffreda D, Gazouli M, Petrone MC, Kelemen D, Ginocchi L, Morelli L, Papiris K *et al.* (2021) Identification of recessively inherited genetic variants potentially linked to pancreatic cancer risk. Front Oncol 11:771312.

16. Purdue MP, Dutta D, Machiela MJ, Gorman BR, Winter T, Okuhara D, Cleland S, Ferreiro-Iglesias A, Scheet P, Liu A *et al.* (2024). Multi-ancestry genome-wide association study of kidney cancer identifies 63 susceptibility regions. Nat Genet 56:809–818.

17. Hughes O, Bentley AR, Breeze CE, Aguet F, Xu X, Nadkarni G, Sun Q, Lin BM, Gilliland T, Meyer MC *et al.* (2024) Genome-wide study investigating effector genes and polygenic prediction for kidney function in persons with ancestry from Africa and the Americas. Cell Genom 4:100468.

18. Scliar MO, Sant'Anna HP, Santolalla ML, Leal TP, Araújo NM, Alvim I, Borda V, Magalhães WCS, Gouveia MH, Lyra R *et al.* (2021) Admixture/fine-mapping in Brazilians reveals a West African-associated potential regulatory variant (rs114066381) with a strong female-specific effect on body mass and fat mass indexes. Int J Obes 45:1017–1029.

19. Secolin R, Gonsales MC, Rocha CS, Naslavsky M, De Marco L, Bicalho MAC, Vazquez VL, Zatz M, Silva WA, and Lopes-Cendes I (2021b) Exploring a region on chromosome 8p23.1 displaying positive selection signals in Brazilian admixed populations: Additional insights into predisposition to obesity and related disorders. Front Genet 12:636542.

20. Chen J, Spracklen CN, Marenne G, Varshney A, Corbin LJ, Luan J, Willems SM, Wu Y, Zhang X, Horikoshi M *et al.* (2021) The trans-ancestral genomic architecture of glycemic traits. Nat Genet 53:840–860.

21. Qu H-Q, Connolly JJ, Kraft P, Long J, Pereira A, Flatley C, Turman C, Prins B, Mentch F, Lotufo PA *et al.* (2023) Trans-ethnic polygenic risk scores for body mass index: An international hundred K+ cohorts consortium study. Clin Transl Med 13:e1291.

22. Downie CG, Shrestha P, Okello S, Yaser M, Lee HH, Wang Y, Krishnan M, Chen HH, Justice AE, Chittoor G *et al.* (2025) Trans-ancestry genome-wide association study of childhood body mass index identifies novel loci and age-specific effects. HGG Adv 6:100411.

23. Carpena MX, Sanchez-Luquez K, Xavier MO, Santos IS, Matijasevich A, Wendt A, Crochemore-Silva I and Tovo-Rodrigues L (2025) Accelerometer-derived sleep metrics in adolescents reveal shared genetic influences with obesity and stress in a Brazilian birth cohort study. Sleep 48:zsae256.

24. Gouveia MH, Cesar CC, Santolalla ML, Anna HPS, Scliar MO, Leal TP, Araújo NM, Soares-Souza GB, Magalhães WCS, Mata IF *et al.* (2019) Genetics of cognitive trajectory in Brazilians: 15 years of follow-up from the Bambuí-Epigen cohort study of aging. Sci Rep 9:18085.

25. Hofer E, Roshchupkin G V., Adams HHH, Knol MJ, Lin H, Li S, Zare H, Ahmad S, Armstrong NJ, Satizabal CL *et al.* (2020) Genetic correlations and genome-wide associations of cortical structure in general population samples of 22,824 adults. Nat Commun 11:4796.

26. Grasby KL, Jahanshad N, Painter JN, Colodro-Conde L, Bralten J, Hibar DP, Lind PA, Pizzagalli F, Ching CRK, McMahon MAB *et al.* (2020) The genetic architecture of the human cerebral cortex. Science 367:eaay6690.

27. McCormack M, Gui H, Ingason A, Speed D, Wright GEB, Zhang EJ, Secolin R, Yasuda C, Kwok M, Wolking S *et al.* (2018) Genetic variation in CFH predicts phenytoin-induced maculopapular exanthema in EUR-descent patients. Neurology 90:e332–e341.

28. Abou-Khalil B, Auce P, Avbersek A, Bahlo M, Balding DJ, Bast T, Baum L, Becker AJ, Becker F, Berghuis B *et al.* (ILAE, 2018) Genome-wide mega-analysis identifies 16 loci and highlights diverse biological mechanisms in the common epilepsies. Nat Commun 9:5269.

29. Kaibara FS, de Araújo TK, Araújo PAORA, Alvim MKM, Yasuda CL, Cendes F, Lopes-Cendes I and Secolin R (2021) Association analysis of candidate variants in admixed Brazilian patients with genetic generalized epilepsies. Front Genet 12:672304.

30. Stevelink R, Campbell C, Chen S, Abou-Khalil B, Adesoji OM, Afawi Z, Amadori E, Anderson A, Anderson J, Andrade DM *et al.* (2023) GWAS meta-analysis of over 29,000 people with epilepsy identifies 26 risk loci and subtype-specific genetic architecture. Nat Genet:1471–1482.

31. Loesch DP, Horimoto ARVR, Heilbron K, Sarihan EI, Inca-Martinez M, Mason E, Cornejo-Olivas M, Torres L, Mazzetti P, Cosentino C *et al.* (2021) Characterizing the genetic architecture of Parkinson's disease in Latinos. Ann Neurol 90:353–365.

32. Kim JJ, Vitale D, Otani DV, Lian MM, Heilbron K, Aslibekyan S, Auton A, Babalola E, Bell RK, Bielenberg J *et al.* (2024) Multi-ancestry genome-wide association meta-analysis of Parkinson's disease. Nat Genet 56:27–36.

33. Rovira P, Demontis D, Sánchez-Mora C, Zayats T, Klein M, Mota NR, Weber H, Garcia-Martínez I, Pagerols M, Vilar-Ribó L *et al.* (2020) Shared genetic background between children and adults with attention deficit/hyperactivity disorder. Neuropsychopharmacology 45:1617–1626.

34. Camerini L, Martins-Silva T, Rohde LA, Santos IS, Barros F, Genro JP, Ghisleni G, Hutz MH, Oliveira I, Matijasevich A *et al.* (2025) Increasing specificity in ADHD genetic association studies during childhood: Use of the oxytocin–vasopressin pathway in attentional processes suggests a specific mechanism for endophenotypes in the 2004 Pelotas birth (Brazil) cohort. Eur Arch Psychiatry Clin Neurosci 275:1925-1937.

35. Akçimen F, Martins S, Liao C, Bourassa C V., Catoire H, Nicholson GA, Riess O, Raposo M, França MC, Vasconcelos J *et al.* (2020) Genome-wide association study identifies genetic factors that modify age at onset in Machado-Joseph disease. Aging (Albany NY) 12:4742–4756.

36. Trubetskoy V, Pardiñas AF, Qi T, Panagiotaropoulou G, Awasthi S, Bigdeli TB, Bryois J, Chen C-Y, Dennison CA, Hall LS *et al.* (2022) Mapping genomic loci implicates genes and synaptic biology in schizophrenia. Nature 604:502–508.

37. Khor CC, Do T, Jia H, Nakano M, George R, Abu-Amero K, Duvesh R, Chen LJ, Li Z, Nongpiur ME *et al.* (2016) Genome-wide association study identifies five new susceptibility loci for primary angle closure glaucoma. Nat Genet 48:556–562.

38. Secolin R, de Araujo TK, Gonsales MC, Rocha CS, Naslavsky M, Marco L De, Bicalho MAC, Vazquez VL, Zatz M, Silva WA *et al.* (2021a) Genetic variability in COVID-19-related genes in the Brazilian population. Hum Genome Var 8:15.

39. Niemi MEK, Karjalainen J, Liao RG, Neale BM, Daly M, Ganna A, Pathak GA, Andrews SJ, Kanai M, Veerapen K *et al.* (2021) Mapping the human genetic architecture of COVID-19. Nature 600:472–477.

40. Pereira AC, Bes TM, Velho M, Marques E, Jannes CE, Valino KR, Dinardo CL, Costa SF, Duarte AJS, Santos AR *et al.* (2022) Genetic risk factors and COVID-19 severity in Brazil: Results from the BRACOVID study. Hum Mol Genet 31:3021–3031.

41. Pairo-Castineira E, Rawlik K, Bretherick AD, Qi T, Wu Y, Nassiri I, McConkey GA, Zechner M, Klaric L, Griffiths F *et al.* (2023) GWAS and meta-analysis identify 49 genetic variants underlying critical COVID-19. Nature 617:764–768.

42. Diz-de Almeida S, Cruz R, Luchessi AD, Lorenzo-Salazar JM, de Heredia ML, Quintela I, González-Montelongo R, Nogueira Silbiger V, Porras MS, Tenorio Castaño JA *et al.* (2024) Novel risk loci for COVID-19 hospitalization among admixed American populations. Elife 13:RP93666.
